# Development of a 3D Printed Brain Model with Vasculature for Neurosurgical Procedure Visualisation and Training

**DOI:** 10.3390/biomedicines11020330

**Published:** 2023-01-24

**Authors:** Manuel Encarnacion Ramirez, Issael Ramirez Pena, Rossi E. Barrientos Castillo, Albert Sufianov, Evgeniy Goncharov, Jose A. Soriano Sanchez, Manuel Colome-Hidalgo, Renat Nurmukhametov, José Rafael Cerda Céspedes, Nicola Montemurro

**Affiliations:** 1 Department of Neurosurgery, RUDN University, 121359 Moscow, Russia; 2 The Royal Melbourne Hospital, Melbourne, VIC 3000, Australia; 3 Department of Neurosurgery, First Moscow State Medical University (Sechenov University), 121359 Moscow, Russia; 4 Traumatology and Orthopedics Center, Central Clinical Hospital of the Russian Academy of Sciences, 121359 Moscow, Russia; 5 Instituto Soriano de Cirugía de Columna Mínimamente Invasiva at ABC Hospital, Neurological Center, Santa Fe Campus, Mexico City 05100, Mexico; 6 Instituto de Investigación en Salud, Universidad Autònoma de Santo Domingo, Santo Domingo 10014, Dominican Republic; 7 Departamento de Farmacia Galénica, Universidad Complutense de Madrid (UCM), 28040 Madrid, Spain; 8 Department of Neurosurgery, Azienda Ospedaliera Universitaria Pisana (AOUP), University of Pisa, 56100 Pisa, Italy

**Keywords:** simulation, neurosurgery, 3D printing, endoscopic third ventriculostomy, ETV, virtual reality

## Abstract

Background: Simulation-based techniques using three-dimensional models are gaining popularity in neurosurgical training. Most pre-existing models are expensive, so we felt a need to develop a real-life model using 3D printing technology to train in endoscopic third ventriculostomy. Methods: The brain model was made using a 3D-printed resin mold from patient-specific MRI data. The mold was filled with silicone Ecoflex™ 00-10 and mixed with Silc Pig^®^ pigment additives to replicate the color and consistency of brain tissue. The dura mater was made from quick-drying silicone paste admixed with gray dye. The blood vessels were made from a silicone 3D-printed mold based on magnetic resonance imaging. Liquid containing paprika oleoresin dye was used to simulate blood and was pumped through the vessels to simulate pulsatile motion. Results: Seven residents and eight senior neurosurgeons were recruited to test our model. The participants reported that the size and anatomy of the elements were very similar to real structures. The model was helpful for training neuroendoscopic 3D perception and navigation. Conclusions: We developed an endoscopic third ventriculostomy training model using 3D printing technology that provides anatomical precision and a realistic simulation. We hope our model can provide an indispensable tool for young neurosurgeons to gain operative experience without exposing patients to risk.

## 1. Introduction

Minimally invasive surgery is gaining popularity in the neurosurgical community [1,2]. Endoscopy within the ventricular system is an accepted form of surgical access for problems such as obstructive hydrocephalus, biopsy, and excision of intraventricular lesions. Endoscopic third ventriculostomy (ETV) is a common surgical procedure used to treat obstructive hydrocephalus. It is one of the most performed procedures in neurosurgery [3,4]. Although minimally invasive, ETV may be associated with significant morbidity and mortality [5,6,7]. The training of surgeons to perform these operations is more challenging than other common neurosurgical procedures given the constraints of access and visualization. Furthermore, most experienced neurosurgeons hesitate to allow supervised training for junior residents because of the relatively small margin of error [8]. Another way to familiarize oneself with the surgical approach, besides performing it on actual patients, is through simulation [9]. Surgical simulators are designed to improve surgical skills while maintaining patient safety [2]. Training in intraventricular endoscopy is particularly challenging due to the small volume of cases and the technical difference from other conventional neurosurgical procedures [8]. Educators can leverage the strengths of medical simulators to effectively teach complex and high-risk surgical procedures without harming the patient [10,11]. Three-dimensional (3D) printing holds promise for expanding access to surgical simulators [12,13,14,15].

Various training models have been used for neurosurgical training, including human cadavers, live animals, virtual reality, and synthetic phantoms [15]. Both open transcranial procedures and minimally invasive neurosurgical procedures have been simulated using these models [1,8,9,10,12,13,14,15,16,17,18]. Animal and human cadaver models offer high fidelity with real patients and allow residents to practice several neurosurgical procedures without the risk of adverse events for the patient. However, they are, of course, expensive, require an anatomical laboratory with specialized facilities and personnel, and in some countries, they are difficult to obtain and raise ethical questions. Finally, human cadaver and animal models lack real tissue properties that closely resemble living human brain tissue and cannot simulate ventriculomegaly [9,10,13,15]. Virtual reality platforms are attractive tools for neurosurgical residents, although they sometimes lack tactile feedback and compelling visualizations and do not allow for the use of real neurosurgical instruments [8,14,15]. Synthetic phantom models, however, are gaining increasing promise as effective simulators for neurosurgical training [12,19,20]. Additive technology, also known as 3D printing, is a novel manufacturing technology that has abundant potential implementations in research and industry [21]. Previous studies reported the use of SLM additive technology to produce structures with specific surface morphological features [22]. According to the literature, phantoms that mimic neurovascular and skull base surgical techniques are common [16]. At the same time, the literature does not provide adequate descriptions of 3D-printed models simulating epilepsy surgery, brain tumor microdissection techniques, and ETV [15,16]. This finding might suggest that 3D printing technology is still in its infancy and cannot yet produce models that can accurately represent fine dissection detail [16]; however, such models are inexpensive, portable, reusable, and permit the use of real surgical instruments. At the same time, they lack realistic tissue properties and can’t be used to simulate surgical complications or complex surgeries. To manage these needs, we developed and here report a novel, reusable, adaptable, interchangeable device with good anatomical details that can be used to improve surgical skills in the use of camera and endoscopic tools as well as in the hand-eye coordination needed to successfully perform an ETV.

## 2. Materials and Methods

### 2.1. Simulator Design and Construction

**Figure 1:**
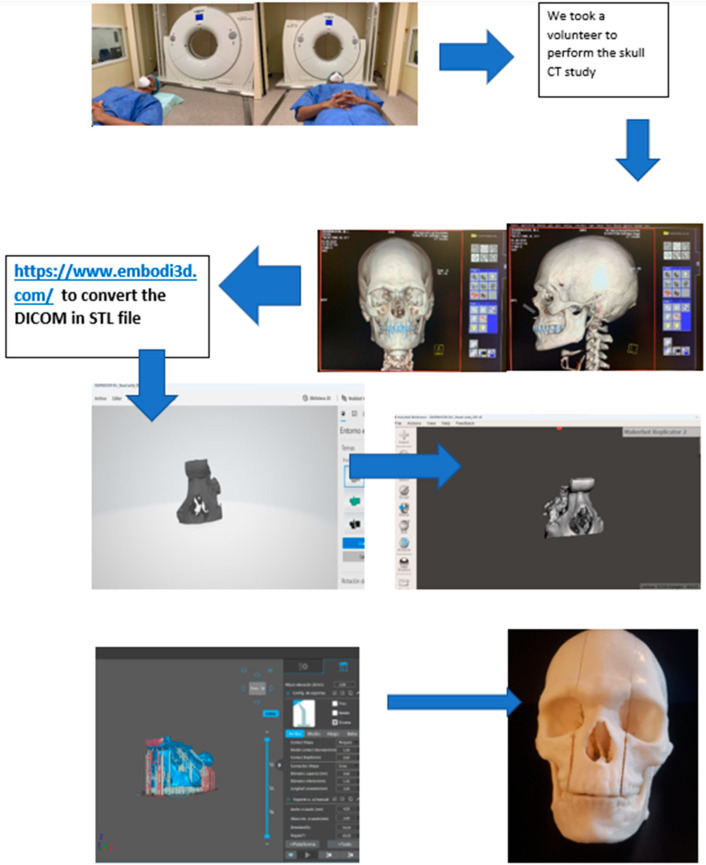
Step 1: perform a CT or MRI scan. Step 2: transform the DICOM files into STL files on a free website (https://www.embodi3d.com (accessed on 25 December 2022)); this can take up to 1 h depending on the DICOM file numbers. Step 3: open the STL files with the Meshmixer program for final processing, repair any errors, and add or remove items. Step 4: open the files with Chitubox for the 3D printer and make the support. Step 5: after the printing process, the phantom is ready.

Elaboration of the skull is made using a human model obtained from a patient-specific set of CT and MRI images. The digital model of a skull is obtained in Digital Imaging and Communications in Medicine (DICOM) format and converted to a stereolithography (STL)-type file. The file is then modified using Meshmixer and is printed in a photopolymer printer, type Kelant S400 using the software CHITUBOX ^®^ V. 1.9 basic. Figure 1 shows the whole production process starting from the CT/MRI scan up to the assembled 3D-printed model.

**Figure 2:**
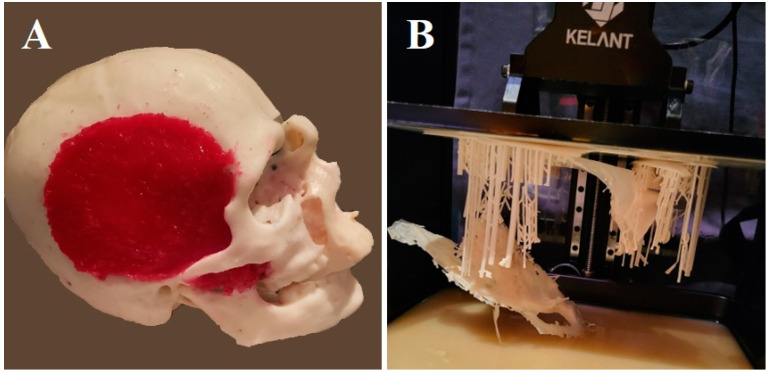
The 3D model (**A**) and supporting material used for printing (**B**).

**Figure 3:**
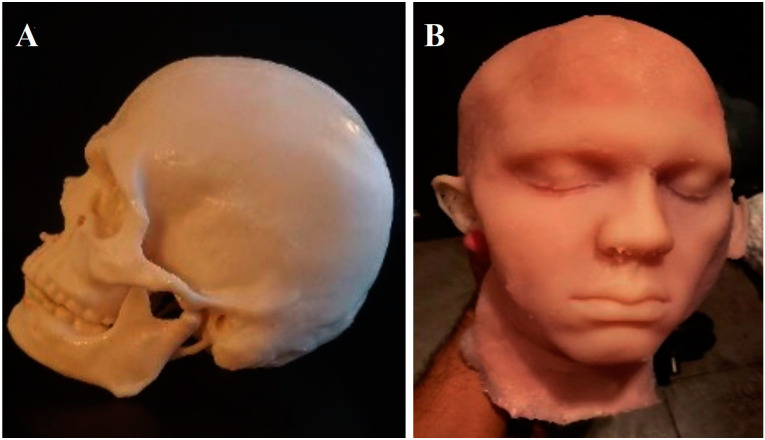
A negative model of the original head is created (**A**). The silicone head looks like an exact copy of the original model (**B**).

After printing (Figure 2), the human skull model is covered using quick-drying silicone, safeguarding the natural holes. The eyebrows and eyelashes are also covered with talc to avoid adhesions. Finally, the model is covered with a cast to give it firmness. In this way, we have a negative model filled with silicone DragonSkin™ Pro and mixed with Silc Pig^®^ pigment additives in a flesh-like color. After the first layer of silicone is cured, we use two layers of silicone DragonSkin™ Pro admixed with Silc Pig^®^ pigment additives in a yellow and red color to give the skin a multilayered appearance (Figure 3).

**Figure 4:**
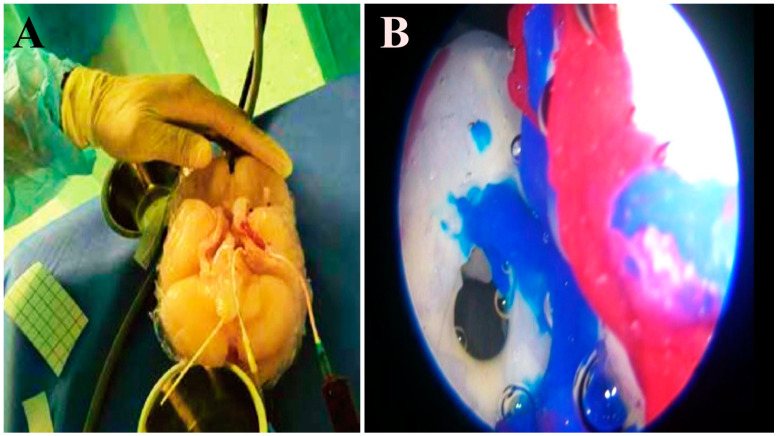
The synthetic dura mater is wrapped around the silicone brain (**A**). The model has a closed-circuit system that provides liquid flow inside the ventricles. Several layers mimic cutaneous, muscular, bony, and dural consistency. Intraventricular view showing foramen of Monro and thalamostriate veins (**B**).

To create the brain, we first obtained a synthetic actual-size model of the brain. Alternatively, an anatomically intact human brain can be used. We used a plastic container leaving a gap of approximately 2 to 3 cm between the brain and the edges of the container. The container was filled with silicone (Tool Decor 25). The brain was then put inside to cure. This formed a “negative” mold of the brain. Once cured, the next step was to remove the brain. The “negative” pieces were filled with silicone Ecoflex™ 00-10 and mixed with Silc Pig^®^ pigment additives in a flesh-like color. Again, we waited until the brain was fully cured (Figure 4A).

We removed the pia mater of the brain as well as all blood vessels. Subsequently, the brain was cut in the midline to get access to the ventricular system. Then, a “negative” mold of the ventricles was constructed. The creation of the ventricular system was a highly complex task, given the anatomical details required. We reproduced the communication between the lateral ventricle and the third ventricle through the Foramen of Monro, as well as the inter-thalamic adhesions (Figure 4B). The floor of the third ventricle was created with a single layer of Tool Decor 25. The mamillary bodies and the infundibulum were added by hand, taking anatomical measurements of the same specimen using silicone type Ecoflex™ 00-30. Thalamostriate veins were made with silicone tool decorator 40 and pigment paste. The accuracy error is close to 2–3 mm, which is similar to other navigation systems or 3D printing.

**Figure 5:**
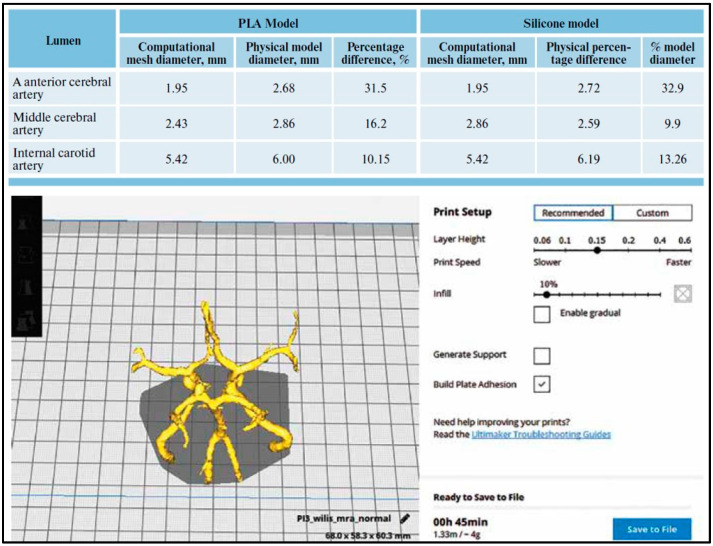
The vasculature surface model edited at the clinical workstation to remove rendering artifacts and crop any undesired vessels.

We used the angiography of a patient after informed consent. We extracted the images in the form of DICOM files and chose the segment needed for the recreation of the cerebral circulation, leaving aside the main vessels. The vasculature surface model was edited at the clinical workstation to remove rendering artifacts and crop any undesired vessels. Figure 5 shows the dimensions of the vessels made and the materials used.

**Figure 6:**
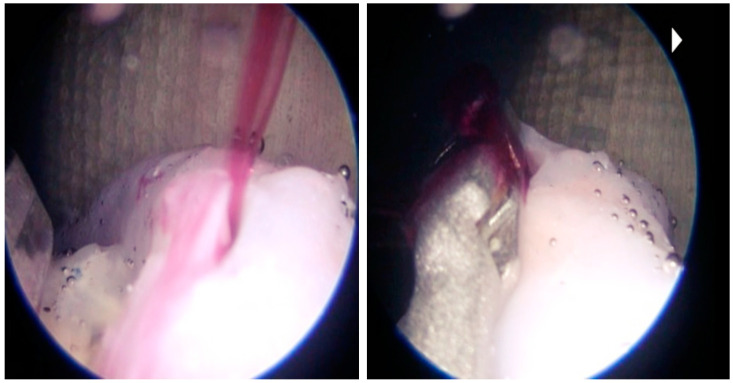
Intraventricular view showing basilar artery bleed.

The vessel model covered with silicone was cut along the length of the vessels to remove the frame. The cut edges of the now hollow vascular structure were sealed with silicone, creating tubular models without leaks. These represent normal vessels. A pulsating pump and liquid dye were then implemented into the blood flow model. In this case, we use liquid mixed with dye paprika oleoresin (E160c). Since the vessels are translucent, it is easy to check for flow, obstruction, or rupture (Figure 6). This dye neither adheres to silicone nor stains the lumen, avoiding any change in appearance. To create the dura mater, we used quick-drying silicone applied with a brush on the entire inner surface of the skull combined with grey polymer “O” PO-MIX dye. All materials have biocompatibility in terms of skin contact.

### 2.2. Cost Analysis

To analyze the manufacturing costs of 3D neurosurgical training models, it is necessary to consider the individual cost of software, hardware, and labor. Energy consumption and other infrastructure costs can be set aside. Our 3D training models were produced with several open-source software applications. For the time of segmentation, modeling in several iterations, and preparation of the prototype model for printing, we estimated approximately 12–25 h of working time; however, the renewed production of the models would now take considerably less time (approximately 10–15 h) due to having an established workflow. All details are shown in Table 1. In addition, as the Kelant S400 3D printer consumes 79 W per hour and the average price of electricity globally is 0.160 USD per kWh, the estimated price of a single skull is about 40 USD in terms of electricity consumption.

### 2.3. Data Collection and Analysis

Eight senior neurosurgeons and seven residents were recruited for the current study. The participants were asked to independently perform an ETV on the simulator. Standard ETV instruments were used, including a rigid endoscope, trocar, grasping and dissecting forceps, and a 4-Fr Fogarty balloon catheter. Videos were recorded during the endoscopic procedures and were stored for editing and analysis. The participants were asked to use our 3D model and give feedback via a survey after the procedure. The survey consisted of the participants rating their level of agreement with each item using a 5-point Likert scale, where: 1 = strongly disagree, 2 = disagree, 3 = neutral, 4 = agree, and 5 = strongly agree. Participants were also asked to comment regarding the simulator’s effectiveness and authenticity as a training tool.

## 3. Results

**Figure 7:**
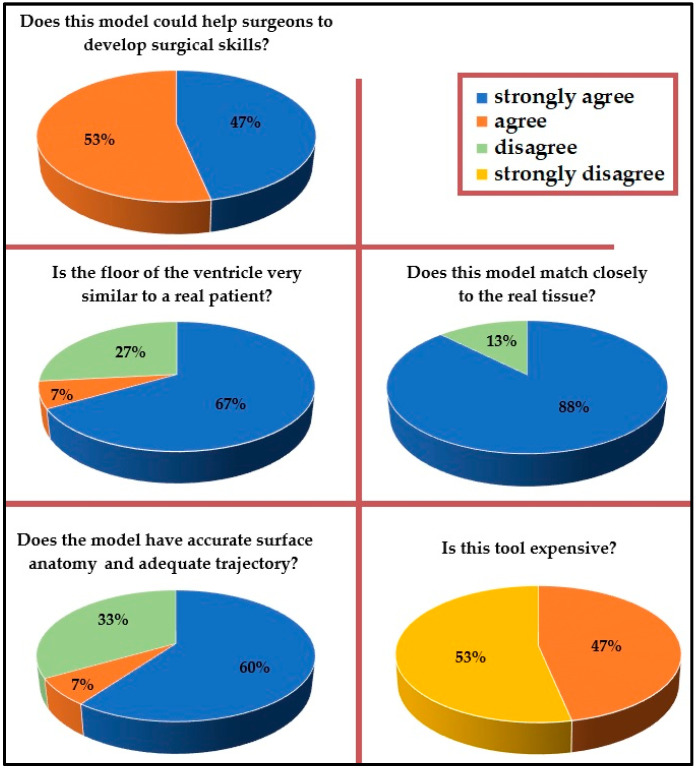
Answers of all neurosurgeons after trying the simulator.

The overall user satisfaction was very high. All neurosurgeons and residents recruited in the current study found our model to be a useful tool for surgical training. Fifty-three percent of them agreed that the simulator could help develop skills in this procedure, and the other forty-seven percent strongly agreed with this statement. Sixty-seven percent of senior neurosurgeons strongly agreed that they would be interested in using this model as a training method. Furthermore, three-quarters of neurosurgeons who had previously performed an ETV before felt the floor of the ventricle to be very similar to a real patient. Sixty-seven percent of participants agreed that the model had accurate surface anatomy, a suitable trajectory, and an appropriately detailed ventricular system. Most of the senior neurosurgeons (87%) agreed that the simulator matches the actual tissue closely. All participants agreed that the use of this model would increase resident competency, hand-eye coordination, and instrument handling. In addition, over half (53%) of the participants considered the model to be an inexpensive tool. Figure 7 reports all neurosurgeons’ answers. Bleeding scenarios were successfully incorporated to subject the trainees to real-life scenarios. Standard neuroendoscopes did not require maintenance or special storage. The production costs were 303 USD for each simulator. The simulator required 25 h to build, with 10 h of labor.

## 4. Discussion

Neuro-endoscopic surgery has a steep learning curve and hence requires continuous hands-on practice [20]. Neurosurgeons began to exploit the potential of the recent technological advancement to improve training [17,23]. Three-dimensional models have proven to be educationally effective [11]. ETV, which is an effective and widely accepted treatment for obstructive hydrocephalus, seemed to be an ideal option for simulation training [12]. There are three basic approaches to medical simulation: (1) cadaveric tissue models (human or animal), (2) computer-based or virtual reality systems, and (3) synthetic physical models [11]. Three-dimensional printed models have proven to be increasingly effective as simulators for neurosurgical training [12,20,23,24,25]. The new generation of 3D printers may enable more professionals to create reliable models providing precise anatomical structures at even lower costs in the future [6,12,26,27,28,29,30,31].

The best surgical training model is the cadaver as it gives the best anatomical picture and feeling. However, not all training centers can easily acquire cadavers given the associated costs and legal implications. Cadaver treatment for training and storage also requires a highly specialized center. During the COVID-19 pandemic, cadaveric practices were considerably reduced, demonstrating that, in the face of adversity, practice on human specimens can be significantly affected. This factor can support the use of 3D models in such situations. The 3D models made from various materials are cost-effective, reusable, and easily replicable. These models can be effectively used up to three times. After the floor of the third ventricle is fenestrated, it can be reconstructed with a layer of silicone. Many such models have been developed for neurovascular simulation, oncology, and spinal procedures. Such simulators offer opportunities to accelerate the development of expertise with respect to new and novel procedures as well as iterate new surgical approaches and innovations, thus allowing novice neurosurgeons to gain valuable experience in surgical techniques without exposing patients to risk of harm [6,32,33,34]. Specific steps in segmentation, processing and the creation of a printable file may impede the workflow or degrade the fidelity of the printed model. Previous papers provided insight into the manufacturing of 3D models of the intracranial vasculature that may facilitate incorporation into or improve the utility of 3D vascular models in clinical practice [32,33]. This 3D simulator model has its own limitations, leaving room for improvement. Even if very similar to actual tissue, this model still lacks the texture of a fresh cadaver. We continue the search for a material resembling the actual brain and vessel texture as closely as possible. Additionally, to create these models, a center needs a high-quality 3D printer and various types of silicone and resin, which are not readily available. A center also needs to have access to CT scans or MRI images and programs to transfer data to a 3D printer for processing. Some steps in the creation of the models are difficult and would require expertise and a training curve. However, once this experience is gained, these models are easily replicable and can be a possible game changer in training programs.

## 5. Conclusions

This 3D simulator helped the residents and senior neurosurgeons to increase their familiarity with the camera skills, instrument handling, and hand-eye coordination required to successfully perform an ETV. It is composed of an actual size head with skin composed of two closed systems, one for the movement of cerebrospinal fluid and the other for the circulation of the basilar artery. This model can be reproduced in any neurosurgery department around the world and each model can be used up to three times. After the floor of the third ventricle is fenestrated, it can be easily covered with a layer of silicone. This model ensures an anatomically accurate and simple approach to surgical training for the third ventriculostomy. The development of future iterations will focus on integration with image guidance systems, incorporation of presurgical planning, and design of patient-specific intraventricular landmarks. In addition, models for other age groups and intraventricular pathologies such as tumors or colloid cysts can be developed. We hope that similar models to this 3D model will be widely used in neurosurgical clinical practice as a new tool for young neurosurgeons all over the world and pave the way for the next step in future surgery.

## Figures and Tables

**Table 1:** Cost analysis information.

Detail List	Price (USD)	Quantity
Silicon Ecoflex™ 00-10	44.00	2
Kelant S400S DLP 3D printers 8.9” LCD 2K laser 3D printer UV Resin SLA light-cure	469.00	1
Silicon DragonSkin™	44.00	1
Anycubic photopolymer - universal WHITE resin for 3D printing, 1 LITER	26.00	1
Silicone Tooldecor 25	23.86	1
SILICONE TOOL DECORATOR 40	23.86	1
Silicon paste coating silicone	22.00	1
Silc pink polymer paste pigment	8.00	1
Silc red polymer paste pigment	8.00	1
Silc blue polymer paste pigment	8.00	1
Silc yellow polymer paste pigment	8.00	1
Silc white polymer paste pigment	8.00	1
Automatic Dosing Pump JEBAO Automatic Water Dosing Pump Aquarium Fish Tank Marine Reef Filtration Peristaltic Pump	30.00	1
Plaster Bandage Cast Orthopedic Tape Cloth Gauze	2.00	3

## Data Availability

The data presented in this study are available on request from the corresponding author.
